# Revisiting episodic-like memory in scrub jays: Is there more we can still learn from what–where–when caching behaviour?

**DOI:** 10.3758/s13420-024-00665-w

**Published:** 2025-01-08

**Authors:** Ella Worsfold, Nicola S. Clayton, Lucy G. Cheke

**Affiliations:** Department of Psychology, University of Cambridge, Cambridge, UK

**Keywords:** Scrub-jay, Episodic-like memory, What-where-when, Corvid, Caching

## Abstract

Professor Nicola Clayton is perhaps best known for her work on food-caching scrub jays. Her seminal 1998 paper, together with Anthony Dickinson, showed that scrub jays could remember what food they had cached, where and how long ago, suggesting memory ability that is ‘episodic-like’ in nature. Here, we present data from a previously unpublished study that sought to replicate and extend these findings. The results replicate previous findings and address potential alternative explanations for earlier results. We argue that the controlled behavioural analyses introduced in this study have the potential to add nuance to our understanding of memory in scrub jay cache retrieval, and to inspire new studies exploring this phenomenon, about which we still have so much to learn.

**Supplementary Information:**

The online version contains supplementary material available at 10.3758/s13420-024-00665-w.

## Introduction

After a century of scientific research into animal intelligence, it is now widely accepted that certain avian species may be capable of cognitive abilities which were previously considered uniquely human. Professor Nicola Clayton has contributed to this field in numerous ways, not least with her work on members of the corvid family, which includes the rooks, ravens, crows, magpies, and jays (e.g., Cheke et al., 2011; Correia et al., 2007; Ostojic et al., 2013; Seed et al., 2006, 2008). One area of particular focus in her work, and that of many others, has been on episodic memory, which refers to the encoding and recall of personal experiences (Tulving & Donaldson, 1972).

In the investigation of episodic memory in nonhuman animals, food-caching birds have been of particular interest, with findings that scrub jays are capable of flexibly using past experiences to shape current behaviour (Clayton & Dickinson, 1998, 1999a; Clayton et al., 2001a, 2001b; Griffiths et al., 1999). Specifically, these birds can recall the *what*, *where*, and *when* (WWW) of caching episodes and use this, alongside knowledge of perishability rates, to inform cache recovery strategies at varying intervals (Clayton & Dickinson, 1998). This integrated *WWW* memory was termed by Clayton and Dickinson as ‘episodic-like memory’ (Clayton & Dickinson, 1998), in recognition of the fact that behavioural tasks can never speak to the phenomenological experience of remembering (or ‘autonoetic consciousness’; e.g., Clayton et al., 2000; Griffiths et al., 1999), which many consider definitive of human-like episodic memory (e.g., Tulving, 1985, 2005). Since its conception, the WWW criteria has been utilised in the study of numerous species, from rats to cuttlefish to apes (e.g., Babb & Crystal, 2006; Davies et al., 2022; Hamilton et al., 2016; Jozet-Alves et al., 2013; Martin-Ordas et al., 2010; Schwartz et al., 2005; Zinkivskay et al., 2009), including humans (e.g., Cheke, 2016; Cheke & Clayton, 2013, 2015; Davies & Clayton, 2024; Hayne & Imuta, 2011; Holland & Smulders, 2011; Newcombe et al., 2014). However, it has often been difficult to establish performance that cannot be explained by alternative explanations, such as associative learning or spatial memory. Food-caching birds continue to provide some of the most concrete nonhuman examples of episodic-like memory, in part due to the opportunity to take advantage of a naturally occurring behaviour that facilitates experimental manipulation of item identity, location, and time delay. The continuous refinement of experiments in response to criticism has strengthened the validity and reliability of these conclusions (e.g., Clayton & Dickinson, 1999a, 1999b, 1999c; Clayton et al., 2001a, 2001b, 2003a, 2003b; Griffiths et al., 1999).

In Clayton and Dickinson’s (1998) ground-breaking original experiment, scrub jays were provided with two ice-cube trays. In Caching Trial 1, one tray was covered (and thus inaccessible) and the birds were given the opportunity to cache one type of food (peanuts or waxworms) in the remaining tray. There was then a 120-h delay, and the birds were given the opportunity to cache the other type of food in the other tray. There was a final 4-h delay and the birds were given access to both trays to recover their caches. During the training trials, the birds discovered that both foods were still palatable after a 4-h delay (and so they would preferentially recover their preferred food, waxworms), but after a 124-h delay the waxworms were rotten, and so only peanuts should be retrieved. When tested in extinction, it was found that the birds searched in the waxworm tray after the short delay, but in the peanut tray following the long delay, suggesting they remembered what they had cached where, and how long ago. Importantly, this cache-recovery behaviour only applied to birds who had had the experience of food degrading. This behaviour meets Clayton et al., and and’s (2003a, 2003b) criteria for episodic-like memory because it required the birds to integrate information about the location and age of a specific food. It also required this information to be used flexibly: the birds could not know, at the time of caching, how long they were going to be away, and therefore they had to assess the age of the cached food *at the time of retrieval* to guide their searching decisions.

Later studies varied details of the design to rule out simple explanations and demonstrate the robustness of the effect. For example, these experiments were able to rule out an explanation of ‘what’ in terms of a preference for one food over another as opposed to a memory for the specific contents of caches (Clayton & Dickinson, 1999a), an interplay between a compulsion to cache irrespective of outcome and a goal-directed motivation to recover specific food caches (Clayton & Dickinson, 1999b). Further experiments ruled out simpler accounts of ‘when’ in terms of relative familiarity as opposed to recalling the passage of time (Clayton & Dickinson, 1999c). They also demonstrated that the birds could keep track of *which* worms had been cached where—and did so in such a way that could only be explained with reference to a flexible integrated representation (Clayton et al., 2001a, 2001b, 2003a, 2003b). Subsequent experiments also showed that the birds remembered the different time profiles of when the various types of perishable foods degraded and that this effect could not be explained by directed forgetting because they could remember with equal accuracy when to search for caches that would ripen over time as well as those that would perish over time (de Kort et al., 2005). Additional experiments also demonstrated that the cache-recovery behaviour of these birds could not be explained purely by associative learning—for example, by contrasting a prediction based on where the birds should search if they associatively or episodically remembered where they had hidden their caches (Clayton, Yu, & Dickinson,2001b, 2003a) and that they did not stop caching in extinction (de Kort et al., 2007).

### Novel measures for old questions

Adapting Clayton and Dickinson’s (1998) methodology, this study aimed to provide a more detailed and robust investigation of episodic-like memory in scrub jays, and address some of the potential alternative explanations that may have applied to previous findings. In particular, we ask whether more nuanced exploration of *what* retrieval behaviours are produced, exactly *where* they are directed, and *when* in the trial they occur, can get a clearly picture of the degree to which scrub jay cache retrieval demonstrates evidence of episodic-like memory.

### Where does searching occur?

One lingering critique of previous explorations of episodic-like memory in scrub jays is that they have employed only two caching trays: One in which the preferred-but-degradable food is cached and one in which the less-preferred-but-lasting food is cached. The issue with this is that, in demonstrating an imbalance in retrieval behaviour between these locations, it is impossible to distinguish between preference *for* a specific location and preference *against* another. Given that food-caching birds are ‘compulsive’ cachers and searchers (e.g., de Kort et al., 2007), jays will rarely not search at all. Thus, if they are avoiding searching in Location A, but only have two locations, they will search in Location B whether or not they expect to find something there. Such a lack of independence between outcome variables makes analysis and interpretation difficult. Some critics (e.g., Suddendorf & Corballis, 2010) argued that results may be explainable through degrading memory trace (but see de Kort et al., 2005). For example, there may be a strong preference for waxworms, leading to stronger location memories for those items, but the accuracy of that memory degrades over time. Consequently, the change in retrieval pattern between short and long delays may be explainable by simple spatial memory which degrades from accurate to inaccurate between the short- and long-delay conditions. If this were the case, one would expect the long-delay condition to be characterised by retrieval behaviours spread across *any* available trays, rather than switching to a specific alternative tray where nonperishable items were stored. To address this and similar accounts, the current study presented birds with four search locations: two that were cached-in and two control trays that were unavailable for caching or searching until the recovery period. This allows not only the independent assessment of the proportion of cache retrieval behaviours in the two cached-in trays (as one is no longer the inverse of the other) but also allows us to distinguish between general tray-directed behaviour (that would be expected across all trays, such as exploration) and behaviour that is more likely to be cache-directed (that would be expected to be specific to trays in which food was cached).

As well as exploring *which tray* searches are directed to, one might also reasonably ask whether searches are accurate *within* a tray. Jays in these experiments tend not to cache in every single cell of the caching tray, meaning that each tray contains both ‘target’ (cached-in) locations and nontarget (uncached-in) locations. Investigating the distribution of searches made across these locations allows several questions to be explored. Most obviously, it allows us to explore whether the jays were precise in remembering exactly where within a tray (rather than ‘in which tray’) they cached. However, it also allows more nuanced investigation; we expect probes made for ‘cache retrieval’ purposes to be more likely to be targeted at cells that were cached in (within a given cached-in tray), while ‘exploratory’ probes might be more evenly spread, or perhaps be more likely to be directed to uncached-in locations (even within the same tray). Following such logic, we might therefore expect spatially accurate searches to be more sensitive to the identity and current desirability of what was cached there, compared with spatially inaccurate searches. We investigate this possibility in two ways. First, we explore whether, when looking only at accurate searches, a higher proportion is made toward the currently edible food, compared with the degraded food. Second, looking at all searches, we explore whether searches in the tray containing the currently edible food are more likely to be accurate, compared with those made in the tray containing the degraded food.

### When does searching occur?

During a 20-min recovery episode, we expect a bird to progress through different types of behaviour, responding to changing motivations including (a) retrieving desired food (i.e., the behaviour of interest), (b) responding to the unexpected absence of caches (perhaps with frustration or searching the nearby area), and (c) engaging in exploratory behaviour, particularly of ‘unexplored’ substrate. Thus, in a caching experiment such as ours, the total number of searches in a given tray over the whole time period may not cleanly reflect only the behaviour of interest, but a whole host of differently motivated actions. Given that we expected cache-retrieval behaviour to be produced first, one way to address this that has been explored in previous studies is to focus on the first search (e.g., Clayton & Dickinson, 1998; Clayton et al., 2001a, 2001b; Marshall et al., 2013). However, this too has its limitations, as it reduces rich behavioural data across an extended time period to a single binary datapoint.

As such, in the current experiment we explored how retrieval behaviour changes across the entire recovery period, focusing on the first probe, the first 3 min, as well as the full episode. While we expected the same overall pattern of search preference regardless of time period, we hypothesised that earlier searches would be more likely to be directed at cached-in trays, while later searches might have higher rates of more exploratory behaviours, spread evenly across trays.

### What search behaviour is produced?

**Fig. 1:**
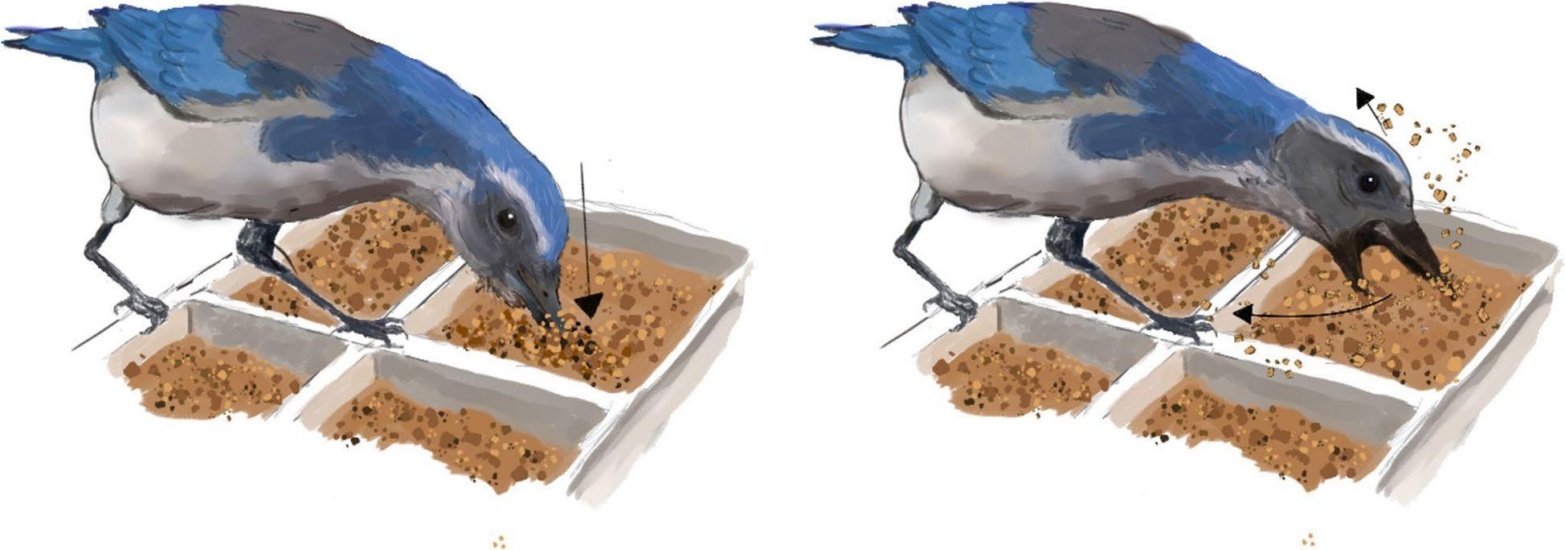
Probe types. Left: A poke directly into the substrate. Right: A swipe across the substrate. Pokes may be indicative of a targeted cache-retrieval attempt, while swipes may be more general search behaviour

Differentiating between exploratory and cache-directed search behaviour may not be achievable only through investigation of *where* and *when* birds search but also in the type of search behaviour they perform. To our knowledge, previous studies of food-caching birds have not differentiated between different types of search behaviours, describing ‘probes’ as ‘any penetration of the substrate surface not involved in general foraging’ (Callo & Adkisson, 2000, p. 87). However, specific types of behaviour have been recorded in passing, noting that subjects often ‘make a sideways sweep of the bill through the substrate with the mandible and maxilla slightly parted’ (Callo & Adkisson, 2000, p. 87). In the coding of behaviour in the current study, we noted a distinction between what we termed ‘pokes’—where the bird directly inserts their beak into a cell of the ice-cube tray—and ‘swipes’—where an open beak is dragged across the surface. ‘Pokes’ appear to pinpoint a specific location in a ‘spear-fishing’ style probe, while ‘swipes’ excavate over a wider area, in a more ‘net fishing’ approach (Fig. 1). Despite being informally noted (Clayton, personal communication), separate recovery behaviours were not distinguished in previous WWW studies. We hypothesise that the ‘poke’ probes are more likely to be performed when the bird is targeting a specific cache in a specific location, whereas ‘swipes’ are more general probing behaviours—perhaps when the cache location is less certain, or when exploring novel substrate. As such, we hypothesised that ‘pokes’ are more likely to show greater sensitivity to what–where–when elements (i.e., sensitive to the location and current desirability of the cache) than swipes, which one might expect to be more evenly distributed. If swipes are indeed more exploratory, one might also expect to see a greater proportion of swipes in the long-delay condition, when memory for the caching event may be more degraded and therefore specific cache location more uncertain.

In summary, this investigation seeks to replicate earlier findings that scrub jays modulate their cache retrieval behaviour based on memories of what was cached, where, and how long ago. We further aim to explore how different types of information—the *what*, *where*, and *when* of the search behaviour itself—can add to our understanding of this behaviour. We hypothesize that behaviours that might be expected to be less exploratory (i.e., targeted pokes, earlier in the recovery period, and those made in cached-in cells) will show greater sensitivity to what–where–when information, and thus provide a cleaner picture of the birds’ episodic-like memory capabilities.

## Methods

Data were collected between September 2013 and October 2014.

### Subjects

**Fig. 2:**
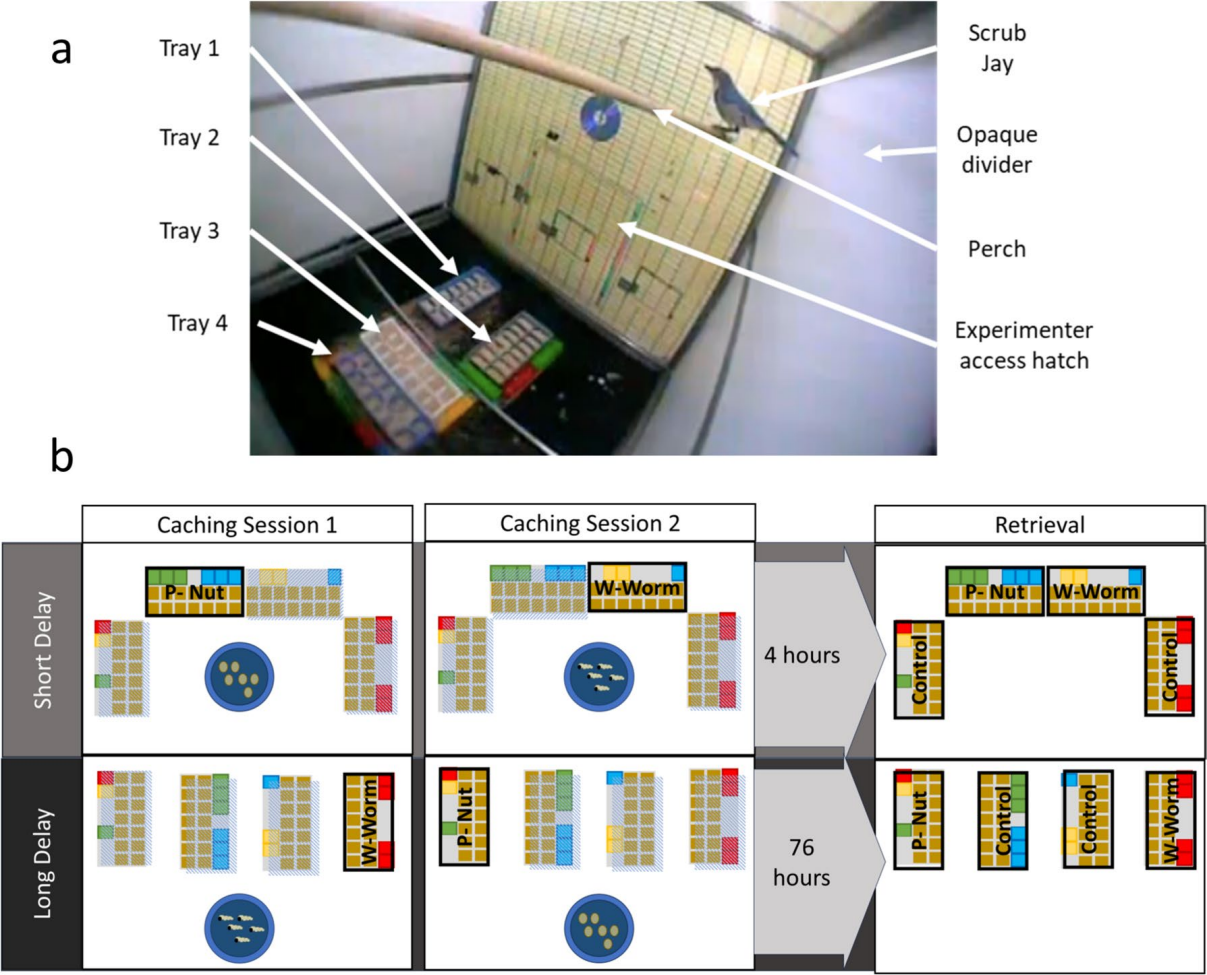
Experimental set up. **a** Photograph of an example retrieval trial. **b** Example tray arrangements, with four caching trays of 14 cells each, and coloured Lego Duplo blocks along the left edge. Accessible trays (for caching or searching) are highlighted in boldface. The position of these trays was varied across trials (but consistent within trials). Birds experienced two caching periods of 15 min consecutively, with the food available counterbalanced across trials. During these, trials only a single tray was accessible (different trays on each period), while two trays remained inaccessible for both periods (control trays). They then experienced a delay of either 4 or 76 h before being allowed to retrieve from all four trays. (Colour figure online)

The study included 11 hand-raised Western scrub jays (*Aphelocoma californica*) of mixed age (7–17 years) and gender, housed at the Sub-Department of Animal Behaviour, University of Cambridge. Jays were housed in indoor cages (2 × 1 × 1 m) within climate-controlled rooms (maintained at 21 °C) and a 12-h light–dark cycle. Each bird was visually distinguished by coloured leg rings and housed with a partner. Their diet, comprising soaked dry cat food, fruit, vegetables, nuts, seeds, and eggs, was removed 2 h before caching and recovery phases. Water was available ad libitum. The testing area ensured visual, but not acoustic, isolation of subjects from their partners to prevent conspecific influence on behaviour. Testing compartments (1 × 1 × 1 m) were created within the standard enclosure using opaque dividers, with one side of each compartment sliding open to allow experimenter access (Fig. 2a). Each compartment was equipped with a wooden perch and a discreet camera to record behaviour. Maintenance, housing and study of these subjects was in accordance with the University of Cambridge and UK Home Office guidelines. Only four birds (two bonded pairs, all aged 7 years) completed training and took part in both test trials [#224 (F), #222 (M), #203 (M), and #207 (F)]. The majority of the analysis is performed with only these birds. All others either did not cache both foods, stopped caching entirely during the training phase, or otherwise did not engage with the experiment. Often, this was due to established and rigid caching preferences, or disinclination to cache in caching trays. Of these other birds, four got far enough through training to undertake one test trial. As this did not allow comparison between delays, these data are not included unless specifically noted but are presented in the Supplementary Materials. Work was conducted under UK Home Office project licence PPL 80/2519. All applicable international, national, and institutional guidelines for the care and use of animals were followed.

### Experimental procedure

Figure 2 illustrates the experimental procedure. During caching, subjects were presented with a bowl containing 40 food items (either pine nuts or waxworms) and four woodchip-filled ice-cube trays (consisting of 7 × 2 cells, each approximately 2.5-cm^2^). Each tray was bordered on one horizontal and vertical side by coloured Lego Duplo™ blocks. This, and the trial-unique tray arrangement, ensured trays were visuospatially unique. Three of the four trays were covered with a clear plastic lid, leaving them visible but inaccessible for caching. Subjects were allowed to cache within the uncovered tray for 15 min before the bowl was removed and the tray covered. The nontray areas of the cage were cleaned of cage caches. A different tray was then uncovered and a bowl containing 40 items of the other food type presented for a second 15-min caching period. After which, subjects were returned to their enclosure and the location, number, and type of caches recorded. The order of food presentation and the position of the four trays was counterbalanced across trials and individuals, but the position of the trays was always consistent within a trial.

Subjects experienced a retention interval of 4 (short condition) or 76 (long condition) hours before returning to the testing compartment for the 20-min recovery phase where all trays were uncovered. Each subject experienced three instances of each delay (4 or 76 h),—namely ‘training trials’, followed by an extinction test trial of each delay, with repeats conducted if the training trial did not offer the appropriate information (e.g., If either food type was not cached). In practice, the four included birds had 6, 14, 8, and 12 training trials in total, respectively, and each bird experienced only one test trial of each delay condition. The order of short- and long-delay conditions was also counterbalanced across subjects, and training of these two periods was conducted in an intermixed fashion. To control for circadian cues, caching and retrieval always occurred at the same time of day regardless of the time delay.

During training trials, caches were replaced with fresh items after short delays, and fresh nuts but degraded worms (blackened to ensure unpalatability) after long delays. The worms were blackened via natural decay: No birds ever ate the blackened worms. This allowed subjects to learn about the relative perishability rates of worms and pine nuts—namely, that worms degrade after longer delays whilst pine nuts do not. Items were replaced in the locations recorded during caching to correspond with the subject’s memory. The woodchip caching substrate was smoothed over to remove visual cues. Test procedure mirrored training, with the exception that caches were removed and not replaced prior to recovery to assess reliance on memory and to control for olfactory cues. Each subject received a single test trial for each delay, and only search behaviour during test trials was analysed. Search behaviours were considered to have occurred if the birds’ beak touched or entered the caching substrate, and there were coded for *what* behaviour was performed (a targeted ‘poke’ or broader open-beaked ‘swipe’; see Fig. 1), *where* the behaviour was performed (in which cell of which tray), and at what time relative to the start of the trial. Note that 100% of behaviours were coded, as all either fell into the category of ‘poke’ or ‘swipe’ and could be attributed an exact time and location.

### Data analysis

Test trials (recovery periods) were recorded and the time, location and type of search behaviour coded by four observers, with 20% overlap. Interobserver reliability was assessed using intraclass correlation coefficients (ICC). This is a method for assessing agreement between multiple coders that emphasises magnitude of difference rather than exact agreement (Harvey, 2021; Koo & Li, 2016). ICC indicated near-perfect agreement (range: 0.90–0.98, where 1 indicates perfect correlation) for total number of searches and good to excellent reliability for the different search types (ICC range: 0.64–0.94).

Given the small sample in this study, most analyses were descriptive, with an emphasis on visual/numerical trends, in recognition that traditional thresholds for statistical significance were unlikely to be achievable. Data were visually inspected via histogram for major deviations from normality and outliers. Statistical analysis was performed using SPSS and R and unless otherwise stated, analysis (including mean values) comprised the four subjects who completed both test conditions (Birds #224, #222, #203, and #207). See the Supplementary Materials for incomplete data on the other subjects. Where appropriate, within-subjects pairwise comparisons were conducted using Friedman’s tests. Between-subjects pairwise analysis was conducted using Wilcoxon; however, given that the lowest achievable *p* value with this sample size is above 0.05 (0.068), this is a reference value only. Multivariate associations and interactions were investigated using a repeated-measures analysis of variance (RMANOVA). For analysis of the first search, binomial and Fishers exact tests were employed.

## Results

### Total number of searches

**Fig. 3:**
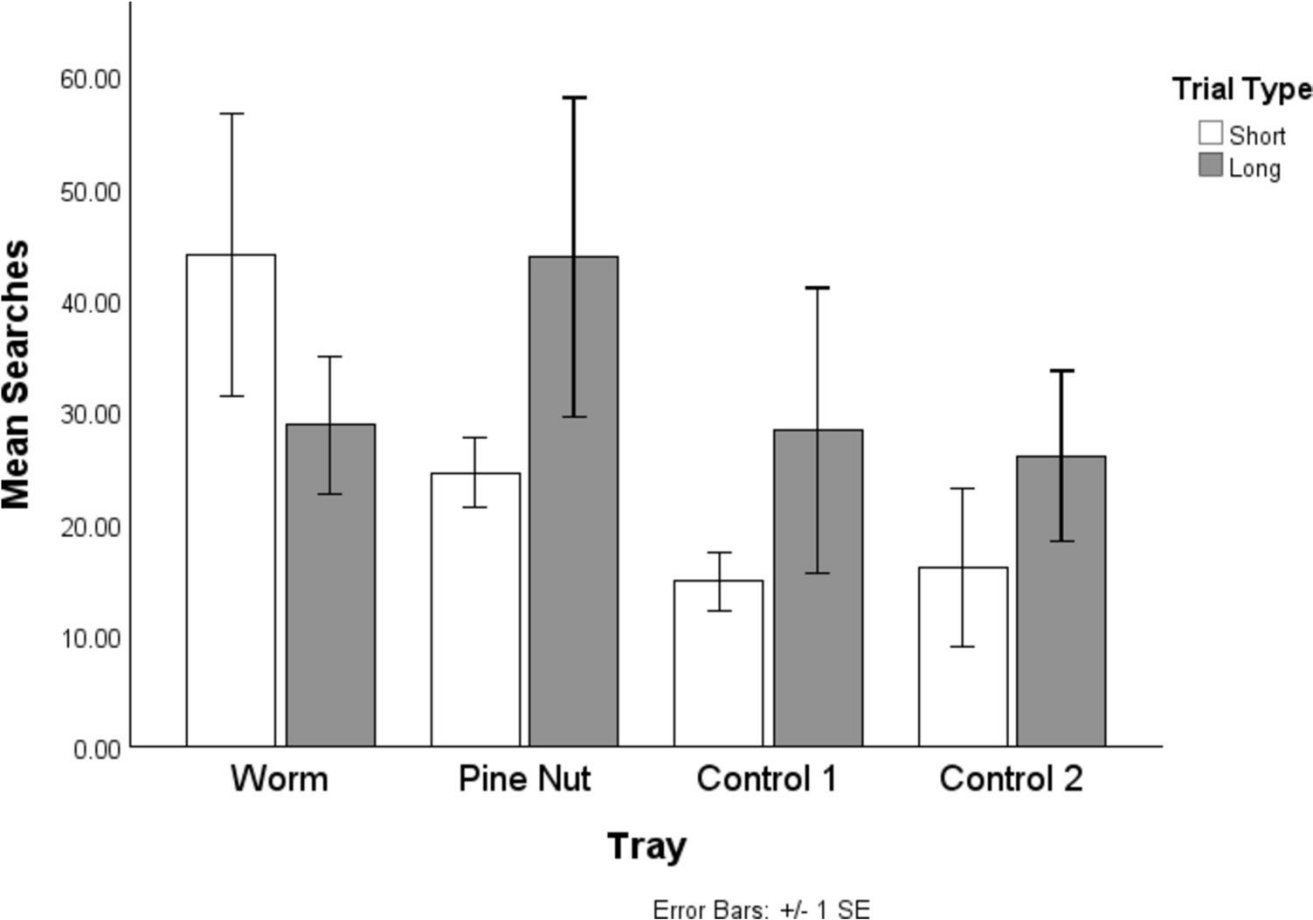
The mean number of searches directed at each tray across the delay conditions’ recovery trials. The most searched tray in the short delay condition was the worm tray, while the most searched tray in the long condition was the pine-nut tray

We first explored the total number of searches made across the entire recovery period. Figure 3 demonstrates the mean number of searches directed at each tray across delay conditions. Visual comparison revealed that numerically more searches were directed at the worm tray in the short condition and the pine nut tray in the long condition. In both delays, the second most investigated tray was the other food-associated one, with the control trays receiving the fewest searches.

Birds searched numerically more in cached-in trays compared with control trays (Fig. 3). This result came close to significance (indeed, as close to significance as is obtainable with the sample): Wilcoxon test, W(1) = 4, *p* = 0.068. A, RMANOVA was conducted to investigate the impact of tray, time delay, and their interaction on number of searches. This was limited to the cached-in trays to maximise degrees of freedom and independence of measurements. There was no significant main effect of tray, *F*(1,3) = 0.263, *p* = 0.643, or delay on number of searches, *F*(1,3) = 0.189,* p* = 0.693, and no significant interaction, *F*(1,3) = 1.798, *p* = 0.272.

### Time analysis

We next explored how search behaviours were distributed over time. Starting with early searches (initial search and first 3 min) followed by analysis of the timeline of searches.

#### Initial searches

**Fig. 4:**
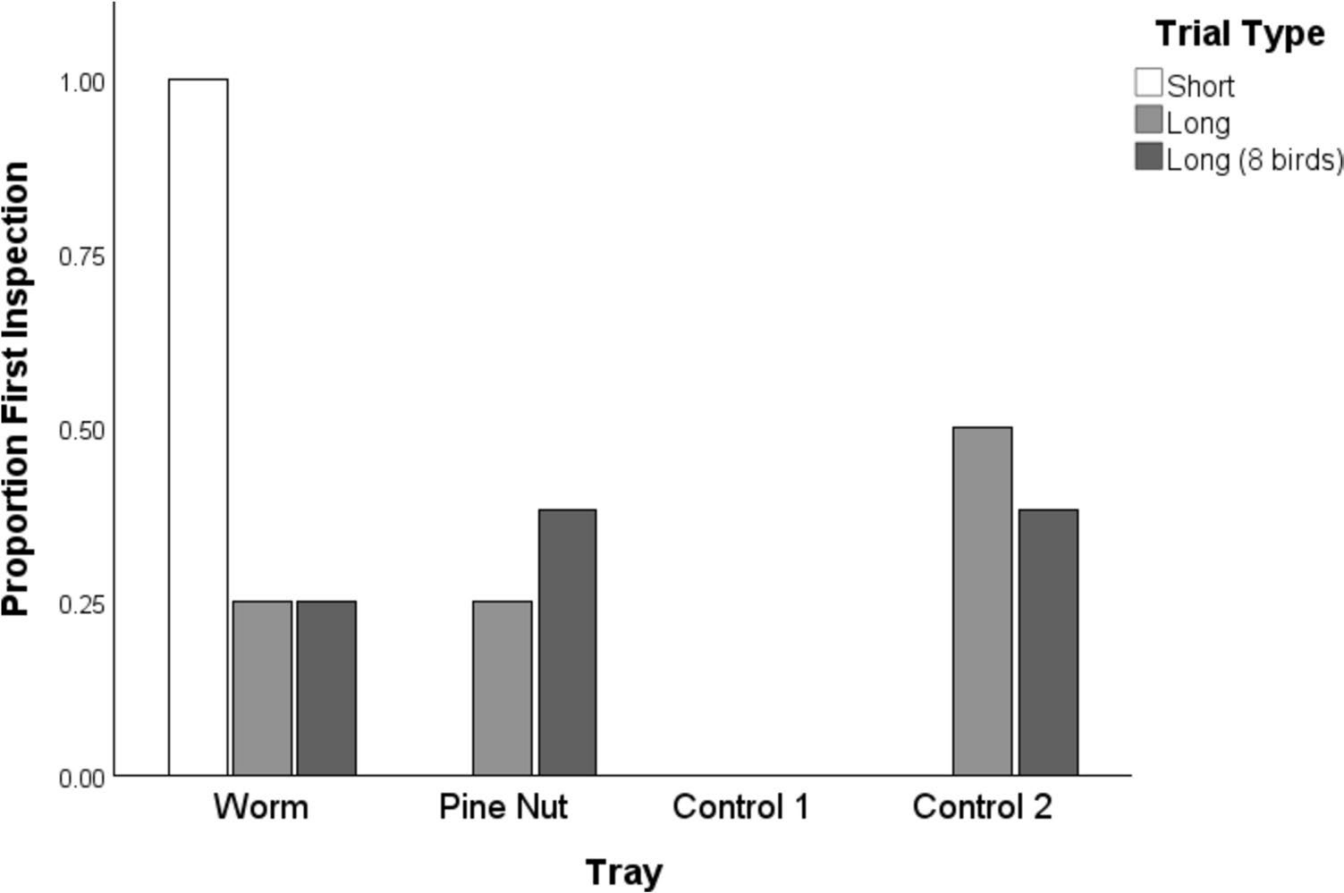
Proportions of initial searches directed at each tray across the short- and long-interval conditions. This includes the four subjects who completed both short- and long-delay tests(short- and long-delay trials) as well as data from four additional birds who only completed the long delay (‘Long (8 birds)’). The worm tray was only searched first in the short-delay test trial, while first searches in the long-delay test trial were distributed across trays

Initial tray selection was assessed using a binomial test. This aimed to determine whether the observed proportion of birds who directed their first search to the worm tray (short condition) and pine nut tray (long condition) significantly differed from chance (0.25). In the short recovery test, all four subjects directed their initial searches to the tray associated with worms (see Fig. 4), significantly deviating from chance expectations (*p* < 0.001). On the long delay, only one of the four birds directed their first search to the pine-nut tray, equalling chance probability (*p* = 1). However, the same proportion (1/4) directed their search at the worm tray, while the other two birds searched control trays.

An additional four birds completed long-delay test trials (but not short delay), and thus this analysis could be conducted across eight birds. Two of the eight birds directed their first search at the worm tray, three of eight to the pine-nut tray, and three of eight to the control trays. Neither waxworm nor pine-nut searches differed from chance (pine nut, *p* = 0.422; waxworm, *p* = 1).

Assessing only the four birds that completed both test conditions, Fischer’s tests revealed no significant differences in the proportion of first searches directed at the pine nut (*p* = 0.49) and worm (*p* = 0.06) tray between the short and long conditions. However, the worm tray showed a numerical trend (Fig. 4).

#### First 3 min

**Fig. 5:**
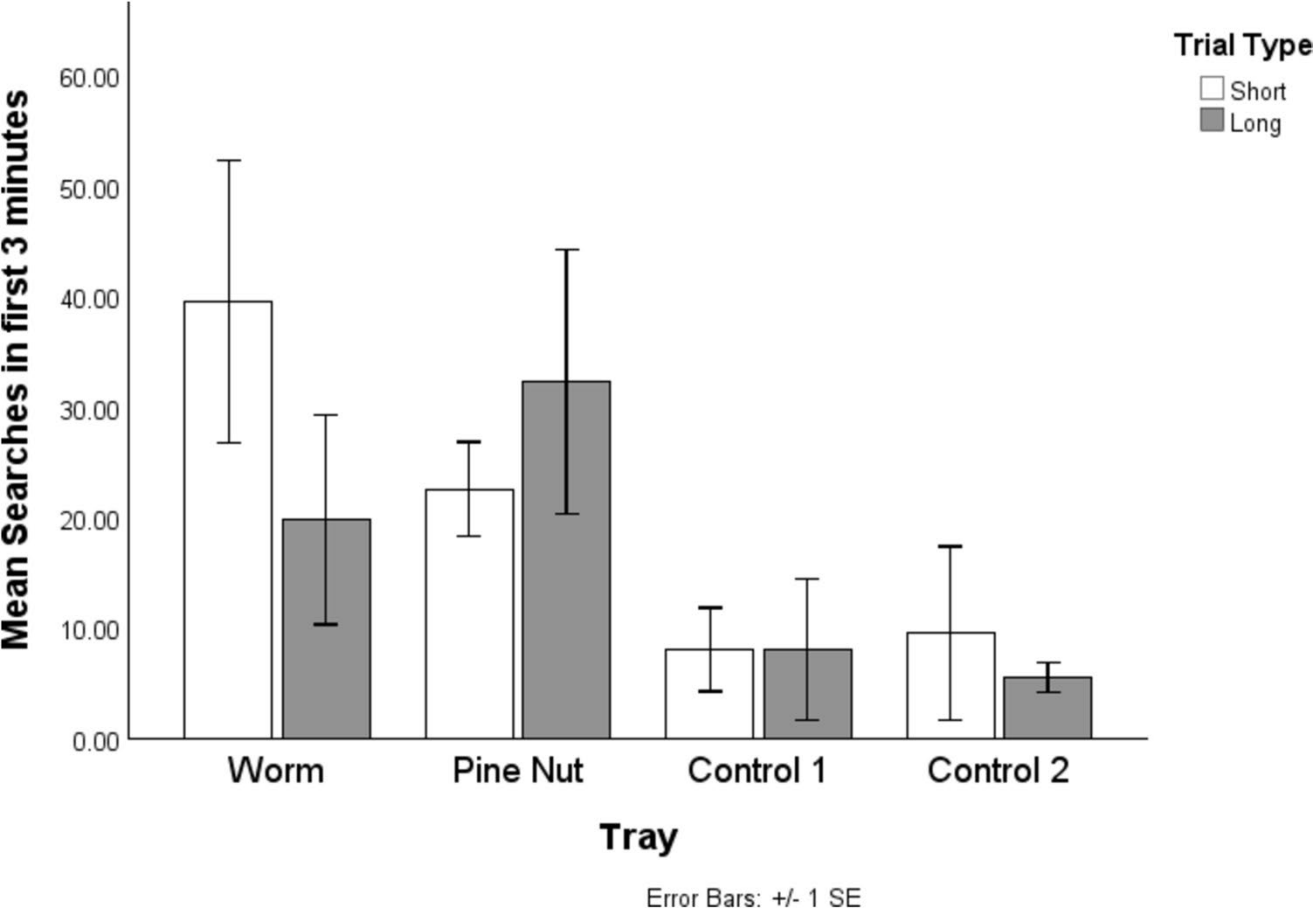
The total number of searches across the first 3 min of the test recovery trials. As with the full-period data, the most searched tray in the short-delay condition as the worm tray, while the most searched tray in the long condition was the pine-nut tray

Birds searched significantly more in cached-in trays compared with control trays in the first 3 min of test trials, Friedman’s test, χ^2^(1) = 4, *p* = 0.046. Figure 5 illustrates the total number of searches across the first 3 min of the test recovery trials. This shows a similar pattern to the total searches, though less clearly. An RMANOVA exploring only the cached-in trays shows similar results, with no significant effect of tray, *F*(1,3) = 0.072, *p* = 0.806, delay, *F*(1,3) = 0.757, *p* = 0.448, or Tray × Delay interaction, *F*(3,1) = 2.229, *p* = 0.232.

#### Timeline

**Fig. 6:**
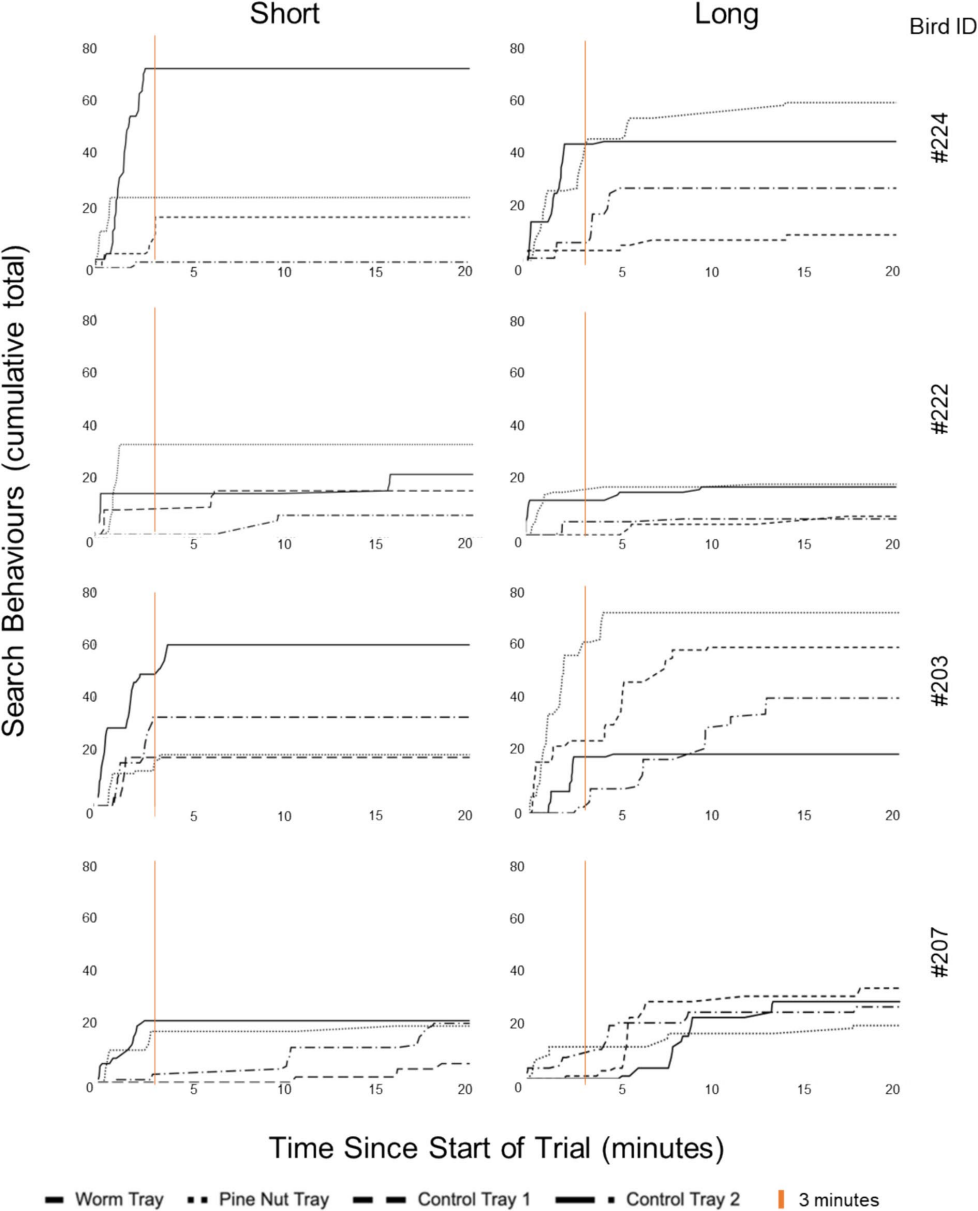
Search allocation across the 20-min recovery period. Bold black lines represent searches in the worm tray, fine dotted lines (which can appear grey) represent searches in the pine-nut tray. Dashed and dash-dot lines represent searches in the control trays

The cumulative searches directed at each tray within test trials were visualised via individual subject graphs (Fig. 6). In short-delay trials, the worm and pine nut-tray searches exhibit a rapid initial increase followed by an early plateau. Typically, the worm tray was searched first (3/4 subjects; excluding #224) and has the greatest overall searches (3/4 subjects; excluding #222, where the pine-nut tray receives the most searches). Compared with the food-associated trays, Control Trays 1 and 2 display a slower rate of increase, with a later onset and result in fewer overall searches. In two or four of the birds, the control tray searches plateau early alongside the food-associated trays, whereas in the other two this was more prolonged and plateaus much later in the trial.

In long-delay trials, searches in the worm and pine-nut trays generally exhibited a quick initial increase followed by an early plateau (excluding #207), whilst the searches in the control trays had a later onset, with more gradual increases over time and a later plateau. This pattern is less pronounced than in the short delay. Interestingly, there was no consistent trend in which trays receive the most searches overall, with only two of four subjects directing most searches to the pine-nut tray ( #224 and #222). Additionally, there was individual variation in investigation frequency, with birds #224 and #203 being more active searchers compared with #222 and #207.

### Search type

Next, we explored the number and distribution of the different types of search behaviour; targeted ‘pokes’ versus broad ‘swipes’.

**Fig. 7:**
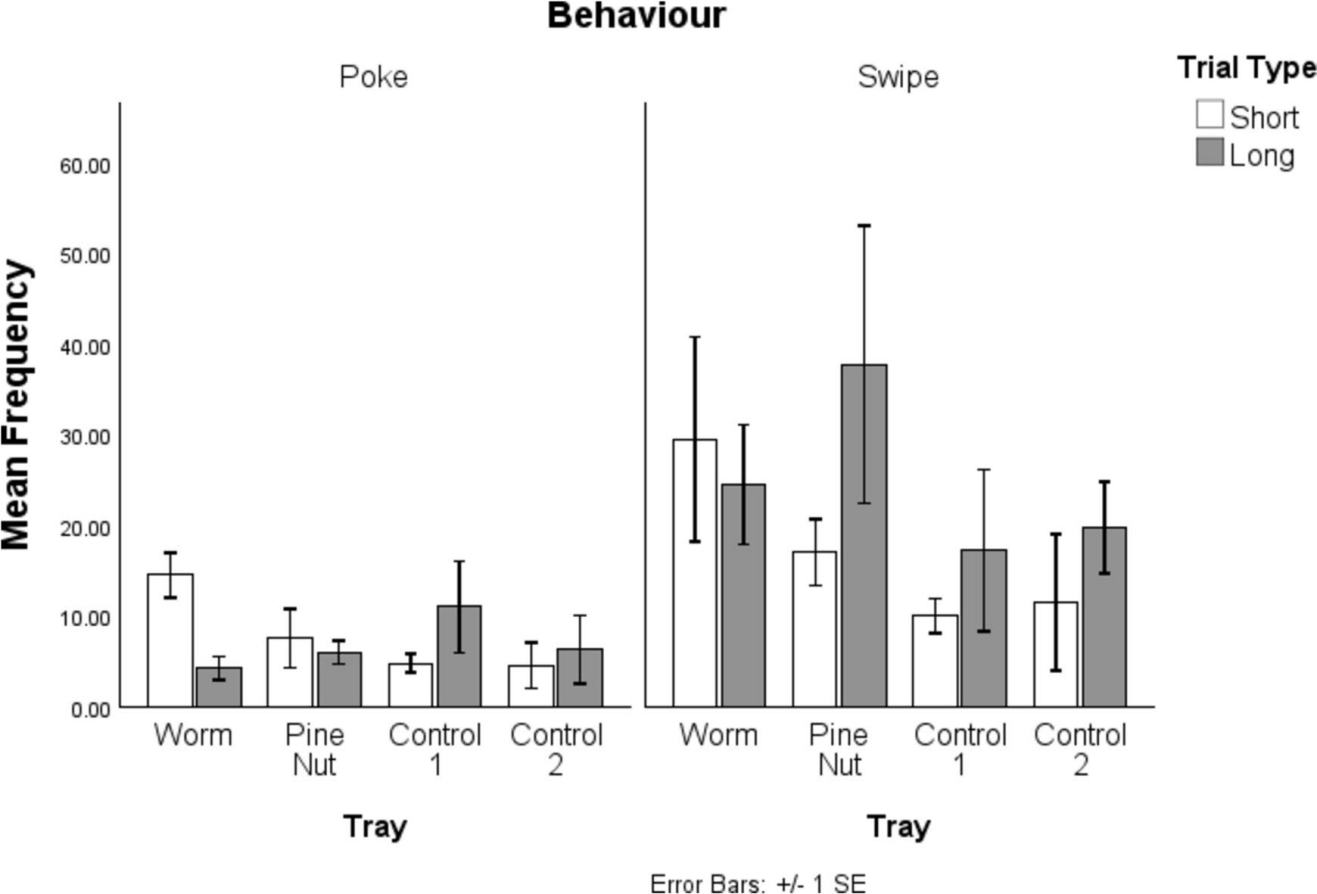
Poke (left) and swipe (right) search behaviours across all four trays and both conditions. There are overall more swipes than pokes, and both show a preference for the worm tray specifically in the short-delay condition

Figure 7 demonstrates the number of pokes and swipes directed at each tray across delay conditions. Numerically, birds consistently produced more swipe probes than pokes (see Table 1) although this was only significant in the long delay condition (*p* = 0.046). An RMANOVA suggested that there was a Probe Type × Delay interaction, with the difference between pokes and swipes being greater in the long-delay condition, Probe × Delay: *F*(1,3) = 23.786, *p* = 0.016. Furthermore, birds searched numerically more (with either search type) in cached-in compared with control trays in both search types, but in neither case did this reach significance, Wilcoxon: poke: W = 1.069, *p* = 0.285; swipe: 1.826, *p* = 0.068.

**Table 1:** Mean and standard deviation for the number of pokes and swipes across each condition, along with RMANOVA test results comparing differences between conditions

	Poke *M* (*SD*)	Swipe *M* (*SD*)	Difference (Poke vs. Swipe) (RMANOVA)
Short delay	31.25 ( 78.96)	68 (27.82)	*F*(1,3) = 5.693, *p* = 0.097)
Long delay	27.5 (18.66)	99.25 (51.54)	*F*(1,3) = 10.534, *p* = 0.048
Total	58.75 (23.49)	167.25 ( 78.96)	*F*(1,3) = 8.406, *p*−0.063
Difference (RMANOVA)	*F*(1,3) = 0.168, *p* = 0.709	*F*(1,3) = 6.231, *p* = 0.088	Interaction (behaviour/delay): *F*(1,3) = 23.786, *p* = 0.016

Separate RMANOVAs were conducted for the different probe types: poke and swipe.

For pokes, an RMANOVA showed no effect of either delay, *F*(1,3) = 2.997, *p* = 0.182, or tray type, *F*(1,3) = 3.349, *p* = 0.165. However, there was a significant Delay × Tray interaction, *F*(3,1) = 40.385, *p* = 0.008, demonstrating a greater preference for the worm tray in the short-delay specifically. There were no significant main effects or interactions for swipes, delay: *F*(1,3) = 2.395, *p* = 0.219, tray type *F*(1,3) = 0.007,* p* = 0.938, Delay × Tray: *F*(3,1) = 0.986, *p* = 0.394.

### Search spatial accuracy

Finally, we investigated the spatial accuracy of searches made, and how these were modulated by current cache desirability. Here, instead of looking simply at the tray that was searched, we looked at the specific cell: Given that not all cells in each tray contained a cache, we can thus compare searches in cached-in cells compared with those made in uncached-in cells. Table 2 shows, accuracy was similar across long and short delays, averaging around 40–50% of searches occurring within cached-in cells. Note that multiple searches in the same cell were often made and each counted in their respective category (accurate/inaccurate).

**Table 2:** Mean and standard deviation for search accuracy across trays and conditions

Condition	Tray	Mean accuracy (%)	Standard deviation (*SD*)
**Short delay**	Worm	53.99	19.36
	Pine nut	39.95	22.50
	**Overall**	**46.97**	**20.83**
**Long delay**	Worm	23.55	29.43
	Pine nut	58.85	18.17
	**Overall**	**41.20**	**29.47**

**Fig. 8:**
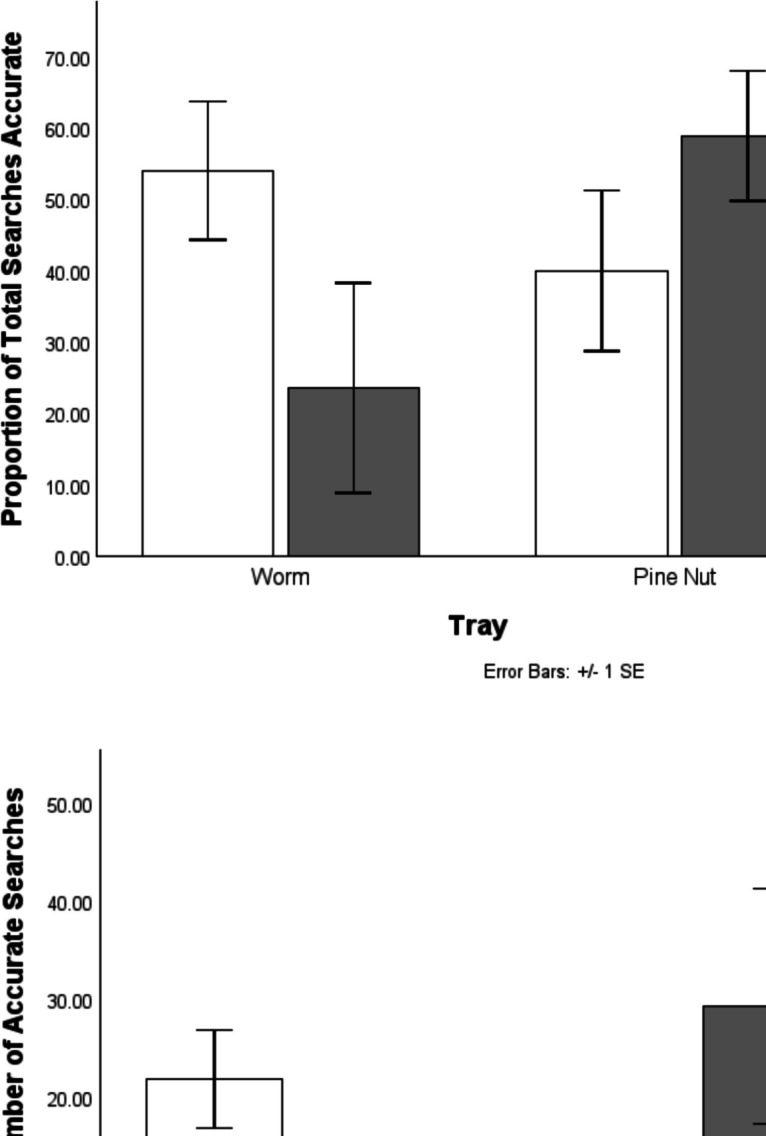
Top: Mean proportion of total searches that were accurate (directed at cached-in cells) in each tray and condition. Bottom: Mean total number of accurate searches in each tray and condition

Visual inspection of the top graph in Fig. 8 suggested that accuracy (correct searches divided by overall number of searches) may vary across conditions and trays. An RMANOVA found no main effect of delay, *F*(1,3) = 0.195, *p* = 0.689, or tray, *F*(1,3) = 0.858, *p* = 0.423. The interaction demonstrated a nonsignificant trend, *F*(1,3) = 9.137, *p* = 0.057. Looking at only correct searches, there was once again no main effect of delay, *F*(1,3) = 0.481, *p* = 0.538, or tray, *F*(1,3) = 0.633, *p* = 0.485. Again, the interaction effect fell short of significance, *F*(1,3) = 7.283, *p* = 0.074.

## Discussion

The data presented here show cache retrieval behaviour of four scrub jays in a what–where–when memory task. The study aimed to replicate previous results (e.g., Clayton & Dickinson, 1998) with additional controls and deeper exploration of the rich behavioural data produced by the birds.

The pattern of results followed the same direction of the previous studies (e.g., Clayton & Dickinson, 1998, 1999a, 1999b), suggesting that the jays searched more for worms in the short condition. We were also able to glean additional insights and greater interpretability from the additional variables explored.

First, our design differed from previous research by the addition of two control trays, increasing the challenge of remembering the location of caches, as well as creating greater interpretability of search behaviour data within the trays of interest: first, by reducing the chance threshold (0.25 rather than 0.5) and second by creating independence in the data, allowing us to distinguish between a choice *for* a given tray from a choice *against* the other. Notably, we see that while the birds did search the control trays, the majority of searches were made within cached-in trays, and these trays were searched earlier in the trial. The greater independence of the data also allows us to observe not only a decrease in preference for the worm tray in the long-delay condition, but a concomitant *increase* in searches in the pine-nut tray. This addresses previous critiques (e.g., Suddendorf & Corballis, 2010) that the change in preference merely reflects decaying memory trace for the preferred food. In particular, when looking at the first 3 min of retrieval, this increase was specific to the pine-nut tray, with no increase in the control trays. Thus, while it is true that general nonworm-tray searching did increase in the long-delay condition, this was initially targeted at the location of the other food.

This study also differed in the dependent variables investigated. In the introduction, we argued that multiple motivations are likely to drive behaviour during a cache recovery period. While we are interested specifically in cache-retrieval behaviour, there will also likely be exploratory behaviour, and perhaps behaviour resulting from confusion or frustration over missing caches (e.g., searching adjacent cells). It can be difficult to differentiate these in order to perform a ‘clean’ analysis of specifically cache-directed behaviour. One traditional way around this difficulty is to focus on only the initial search behaviour (e.g., Clayton & Dickinson, 1998; Clayton et al., 2001a, 2001b; Marshall et al., 2013). However, this is a very conservative measure that reduces 20 min of rich behaviour into a binary data point. Another option is to embrace this richness and investigate different types and patterns of searching.

Analysing the timeline of search behaviour, we saw that the pattern shown in the first 3 min broadly reflected that seen across the entire 20-min period, and that this was also loosely reflected in the first-search data. In all cases, the preference for waxworm tray in the short delay (rather than the long delay) is the most clear, while the preference for pine nuts was more mixed, with the delay conditions proving more even. Visual inspection of the allocation of tray searches over time suggests that the cached-in trays are generally explored earlier (see also Supplementary Fig. 4), while the control trays are paid more attention later in the trial, supporting the suggestion that these later probes are more exploratory. Given this, we might have expected to see a clearer preference for currently edible food in the first 3 min relative to the entire recovery period. It is possible that this was not the case purely due to the relative reduction in data points resulting from focusing on such a short period of time (particularly given the already small sample size). It is also possible that these early minutes, while less likely to contain ‘exploratory’ behaviours, might incorporate the period in which birds are discovering and reacting to the disappearance of their caches (due to the test being conducted in extinction), and may therefore dedicate some search time to adjacent cells and ‘frustration’ behaviours.

Investigating the type of search behaviours made, it was clear that the potentially more exploratory ‘swipe’ behaviours were made more often than the more targeted ‘poke’ probes overall, and particularly in the long delay condition. This distinction may lie in the fact that memory may have degraded more after the longer delay, leading to more uncertainty as to the exact location of caches. Interestingly, while both behaviours appear numerically to show some sensitivity to the what–where–when elements of the task, it was specifically the poke probes that demonstrated a significant effect, with more probes being made in the worm tray in the short condition specifically. This would align with the notion of pokes being more likely to be made as specific cache-retrieval behaviours, and thus more modulated by current reward value of cache content. One way to consider these different behaviours is as a proxy for ‘confidence’. In human memory research a distinction is often made between recognition or recalls that are judged as ‘high confidence’ and those that are ‘low confidence’ or better described as a ‘guess’, with the former being generally more accurate (e.g., Stretch & Wixted, 1998; Yonelinas & Jacoby, 1994). While related to accuracy, memory confidence is separable both behaviourally (Roediger & McDermott, 1995; Schacter & Dodson, 2001) and neurally (e.g., Simons et al., 2010) and can be influenced by multiple factors (e.g., Chua et al., 2012). As such, the ability to distinguish behaviourally those searches that are more ‘confident’ cache retrieval attempts is likely to enrich memory research in food-caching birds.

Finally, we looked at spatial accuracy, both in terms of which trays were more accurately searched (and how this was modulated by delay and cache content), as well as to what extent the spatially accurate searches (where) were modulated by what-when information. This is another approach aimed at pinpointing only those probing behaviours intended as cache retrieval. It is likely that spatially accurate searches (i.e., those made in cells in which a cache was made) are more likely to be attempts at cache retrieval than spatially inaccurate ones, which may be more likely to be a mix of misdirected cache-retrieval attempts and exploration behaviour. As such, you might expect to see greater modulation of accurate searches by cache desirability, compared with analysis of overall searches. On the flip side, it might be expected that a greater proportion of searches in (general) locations where desired food are located will be (specifically) spatially accurate compared with those in (general) locations with less desirable food, as more behaviour in this area is likely to be active ‘cache retrieval’. Both analyses were marginally nonsignificant, with an observable trend suggesting greater spatial accuracy being linked to the waxworm tray specifically in the short-delay condition, and to the pine-nut tray specifically on the long-delay condition.

Overall, it appears that the birds in this study did modulate their search behaviour based on what was cached, where, and how long ago (i.e., what–where–when ‘WWW’ information). This pattern was observable in overall searches, but particularly clear in those behaviours that might be more likely to represent cache-retrieval attempts. Specifically, we show that probes that are directed in a targeted manner (i.e., ‘pokes’) show modulation by WWW information most clearly, and that when probes are made in cached-in cells, they are more likely to be in the cells in which currently desirable food was cached. Finally, we show that search behaviour alters across the recovery time course in a manner suggesting that cache-retrieval searches are likely to be made earlier than more exploratory searches (e.g., in never-cached-in trays). By looking at *what* behaviour was produced *where* and *when*, we were able to get a much clearer picture of the memory capabilities of the jays.

## Summary and conclusions

Twenty-seven years on from Clayton and Dickinson’s original finding of episodic-like memory in jays, the what–where–when paradigm has inspired excellent work in multiple other species and contexts (e.g., Babb & Crystal, 2006; Cheke et al., 2016, 2017; Davis et al., 2013; Hayne & Imuta, 2011; Jozet-Alves et al., 2013; Martin-Ordas et al., 2010; Zinkivskay et al., 2009). However, even returning to the original context of use—cache-retrieval behaviour in scrub jays—the approach continues to offer new insights and inspiration. The work presented here suggests that focusing on times and behaviours more likely to be specifically cache-directed tends to show a greater modulation of scrub jay probing behaviour by current cache desirability (as dictated by what was cached, where, and when). This data provides a proof of concept that including such variables is not only feasible but provides richer, cleaner, and more interpretable data, and as such that they should be utilised in future work. They also provide additional confidence that earlier findings genuinely demonstrate episodic-like memory in scrub jays.

## Supplementary Information

Below is the link to the electronic supplementary material.

Supplementary file1 (DOCX 732 KB)

## Data Availability

The datasets generated during and/or analysed during the current study are available from the corresponding author on reasonable request.

## Code availability

There is no relevant code to be made available.
