# Supplementary material for: Revisiting episodic-like memory in scrub jays: Is there more we can still learn from what–where–when caching behaviour?
Source: Learn Behav. 2025 Jan 8;53(1):65–79. doi: 10.3758/s13420-024-00665-w (PMC11880068; doi:10.3758/s13420-024-00665-w)
Supplement: Supplementary file 1 — Supplementary file1 (DOCX 732 KB) [file 13420_2024_665_MOESM1_ESM.docx]

**Supplementary materials.**

**Appendix 1: Data from additional birds.**

As described in the methods, four additional birds completed the long delay test but not the short delay test condition. The data from these birds is presented below.

*Number of Searches.*

Overall search data from the additional birds is shown in Figure SM1. It is difficult to form strong conclusions based on this incomplete data, however it is notable that the pattern of a greater number of searches in the pine nut tray relative to the wax worm tray is similar to that seen in the birds that completed both session.

Figure SM1: Searches made in each tray made by the four additional birds ( #108; #210, #220 and #229).

*Timing of Searches*

Searches over time for the additional birds is shown in figure SM2. These are similarly heterogeneous as with the birds analysed in the main text. 3 / 4 birds appear to show a very early preference for cached-in trays, but in almost all cases this is quickly supplemented with searches in the control trays. 2 / 4 birds search the pine nut tray before the worm tray.


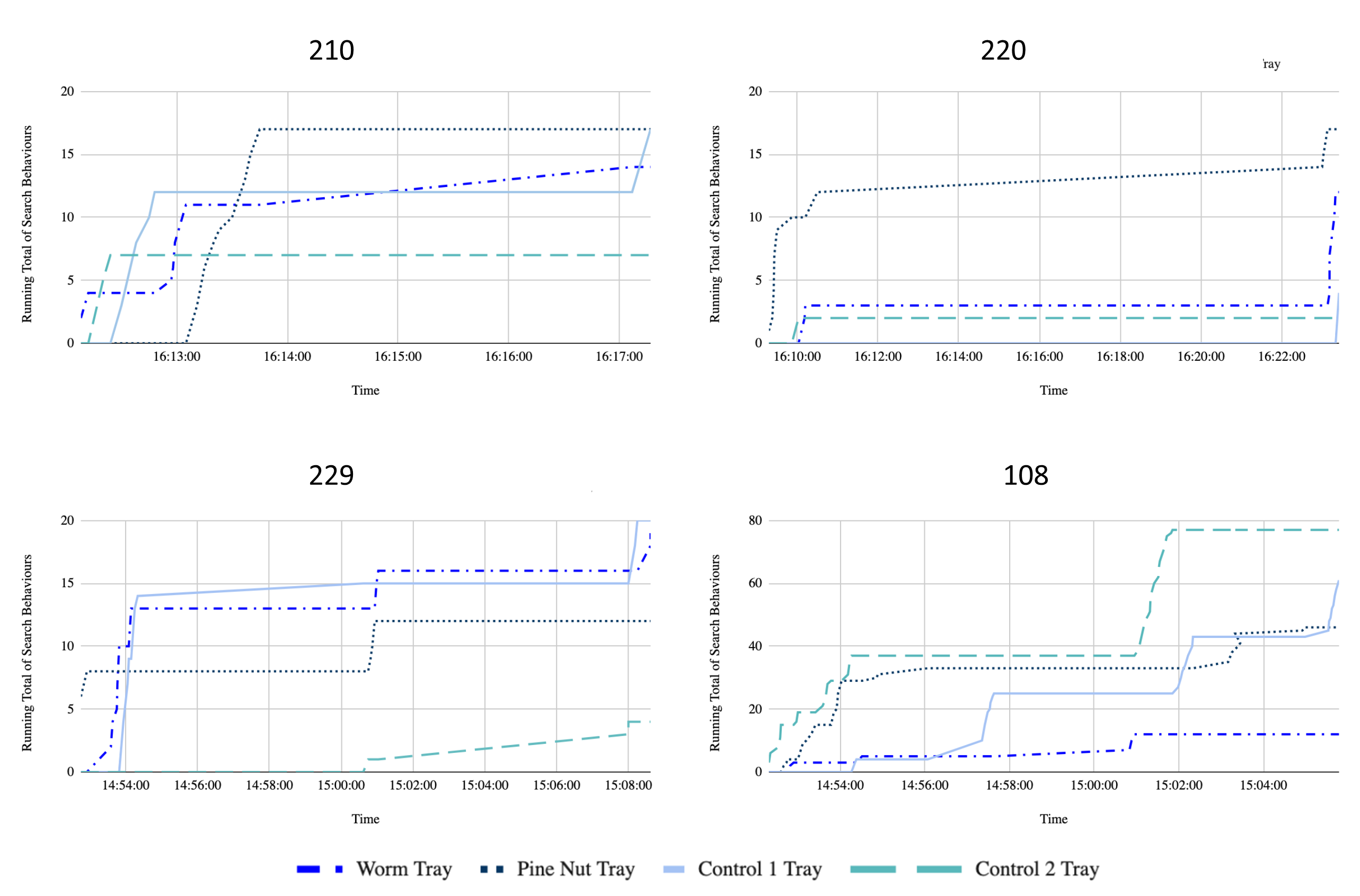


Figure SM2: searches over time in the four additional birds ( #108; #210, #220 and #229).

*Different search behaviours*

Figure SM3 shows the distribution of different search behaviours (poke vs swipe) in the additional four birds. Here we can see a similar pattern of generally more swipes than pokes. We also see that the greater preference for the pine nut tray over the wax worm tray is seen in the pokes but not the swipes.


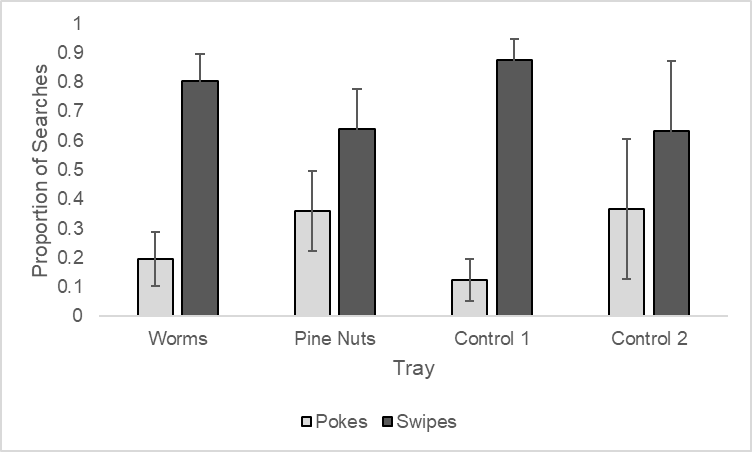


Figure SM3. Proportion of search behaviours that were pokes or swipes across the different trays in the four additional birds.

**Appendix 2: Behaviour over Time – More detail.**

*The last 17 minutes*

The inverse of looking at the first three minutes of a trial is looking at the remaining 17 minutes (in the central 4 birds). Figure SM4 shows the difference between inspections in the first three minutes compared to the entire session. Here we can see that the majority of the additional searches are made in the control trays, and particularly in the long delay condition. This suggests that while the pattern of searches across the cached-in trays was similar between the first three minutes and the overall session, the major difference between these session is the proportion of searches directed at control trays. This supports the concept that a greater proportion of late-trial probes are exploratory rather than motivated by cache-retrieval.


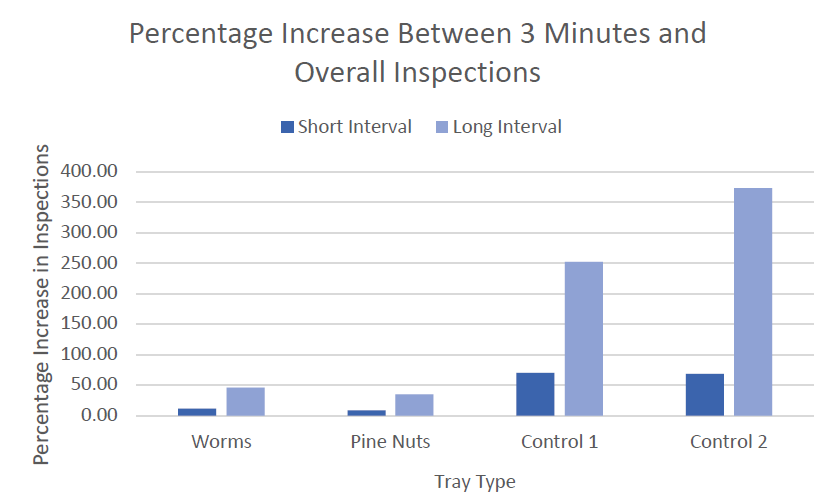


*Figure SM4: Bar graph displaying percentage increase in mean tray inspections between three*

*minutes and overall. Short interval= dark blue, long interval= light blue.*

**Appendix 3: Timing of Different Search Behaviours**

Poke and swipe frequencies may also differ across the recovery period, which would be obscured by overall values. If differentially exploratory or affected by frustration, we would expect more pokes initially, followed by increasing swipes as subjects discover caches have been pilfered. To assess this, cumulative pokes and swipes over the recovery tests (combined across all trays) was visualised for each bird and condition. This includes the four birds who only completed the long interval test. Figures SM5 reveals that in the majority of instances the cumulative swipe frequency is higher than pokes (except #222 short) and that there is a rapid initial increase in behaviours which plateaus around halfway. Beyond this, there was not a shared pattern of relative poke/swipe behaviours across subjects and trials. For example, #203 and #224 short showed a gradual linear increase of both behaviours, before pokes plateaued around halfway. Whilst in other trials, the initial swipe rate was much greater than the pokes, before both plateaued around halfway (#203 long, #222 long, #224 long, #207 short). In others (#222 short, 108 long, #220 long, #229 long), the pattern and frequency of both behaviours were similar throughout.


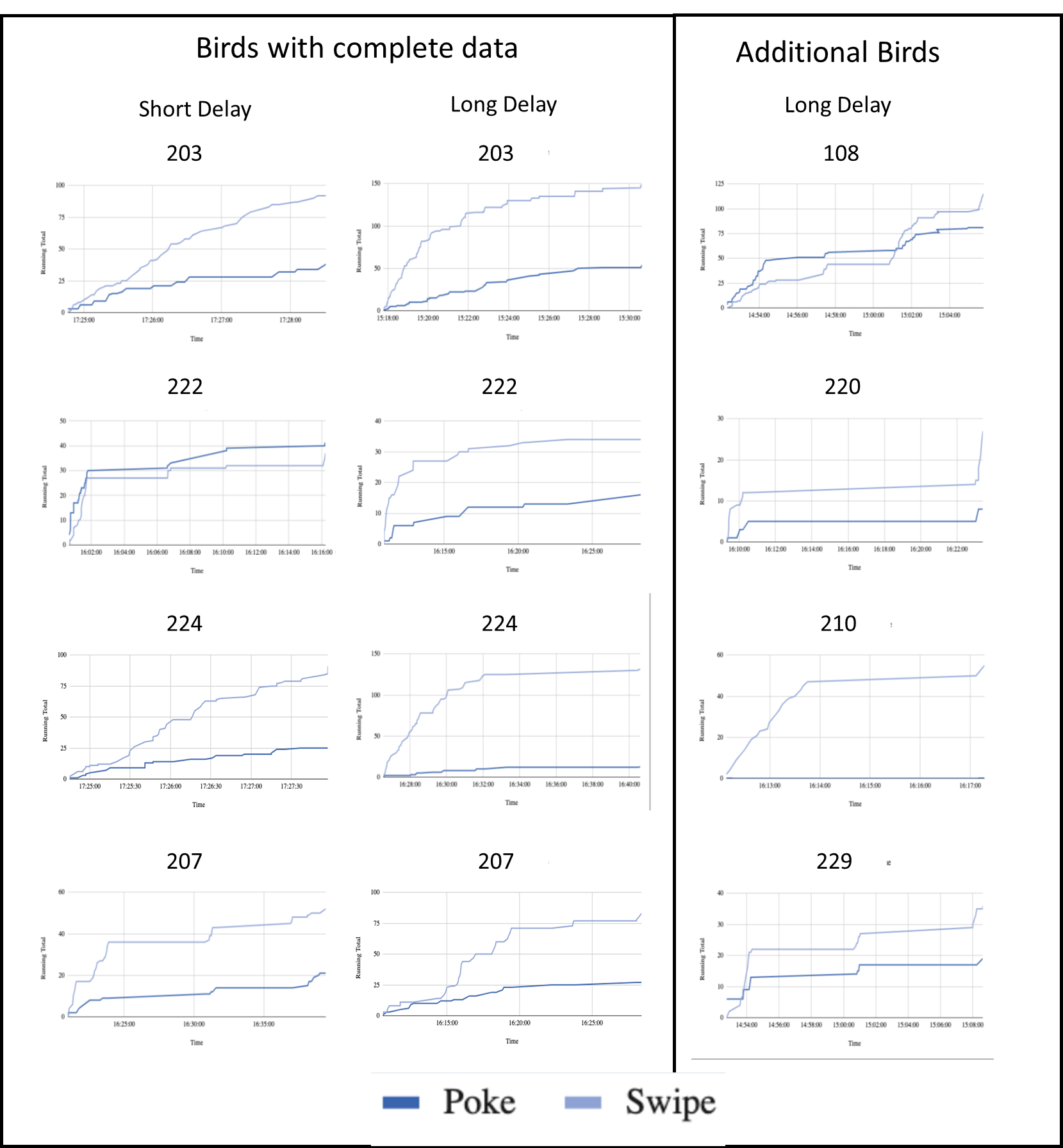


Figure SM5: Distribution of pokes and swipes over time. No clear pattern of one behaviour being produced earlier than the other.
